# Toll-Like Receptor 4 in Inflammation and Angiogenesis: A Double-Edged Sword

**DOI:** 10.3389/fimmu.2014.00313

**Published:** 2014-07-07

**Authors:** Sheeba Murad

**Affiliations:** ^1^Molecular Immunology Lab, Health Care Biotech Department, Atta-Ur-Rahman School of Applied Biosciences, National University of Sciences and Technology, Islamabad, Pakistan

**Keywords:** LPS, TLR4, PAMPS, DAMPs, angiogenesis

Toll-like receptors (TLRs) primarily known for the pathogen recognition and subsequent immune responses are being investigated for their pathogenic role in various chronic diseases. The recent reports correlating the microbial infections with chronic disorders such as atherosclerosis have lead to questions in relation to the role of microbial sensors such as TLR4 in an intriguing phenomenon of the *inflammation-induced angiogenesis*. This article focuses on the possible mechanisms involved in it.

Toll-like receptors comprise a large family of the pathogen-pattern recognition receptors (PPRR) originally identified in *Drosophila* in the mid 1990s as a *Toll protein* (1). In *Drosophila*, it was found to be involved in the resistance against fungal infections (2). The first human homolog for the Toll protein was described in 1997 (3). Since then, 13 mammalian homologs of the TLR family have been identified; including 12 in mice (TLR1-9 and TLR11-13) and 10 in humans (TLR1-10). TLR 10 is a pseudogene in mice, but is functional in humans (4). The membrane expressed TLRs recognize the pathogen-associated molecular patterns (PAMPs) either directly on the plasma membrane or within the endosomal compartment after the phagocytosis. In addition to the foreign molecules, a range of various endogenous ligands are also detected by TLRs, which suggests a role beyond that of simple pathogen recognition. Endogenous ligands released from the damaged, apoptotic, or fibrotic cells during inflammation, are termed *danger-associated molecular patterns* (DAMPs). A significant number of DAMPs have been reported for TLR4 (5, 6).

TLR4 is one of the best characterized and the first member of the TLR family to be discovered as a PPRR. TLR4 signaling is implicated in the innate immune responses against a wide-range of microbes, including Gram-negative and -positive bacteria, mycobacteria, spirochetes, yeasts, and some viruses such as respiratory syncytial viruses (RSV) and mammary tumor viruses (4). TLR4 is a type I transmembrane protein characterized by an extracellular domain containing leucine-rich repeats (LRRs) and a cytoplasmic tail harboring a conserved region known as Toll/IL-1 receptor (TIR) domain. TLR4, along with its two co-receptors, the myeloid differentiation antigen (MD2) and the LRR protein CD14, forms a trimeric receptor that is involved in the recognition of lipopolysaccharide (LPS). The TLR4 ligand binding causes the C termini of the ectodomains to move close to each other, thus triggering signaling and inflammation. The diverse interactions between TLRs with their ligands converge into either the MyD88-dependent or MyD88-independent pathways, resulting in the: (1) activation of lymphocytes, (2) up-regulation/expression of co-stimulatory signals, and (3) release of pro-inflammatory cytokines/chemokines (7). As sentinels in the innate immunity, TLR expression was thought to be confined to the immune cells such as macrophages, monocytes, and dendritic cells. However, an increasing number of reports show a more diverse expression of TLRs; including epithelial cells, endothelial cells (8), neural and glial cells, thereby playing an important role in tissue-specific inflammation (9).

TLR4 is implicated in a diverse range of pathological processes associated with or induced by angiogenesis including autoimmune diseases such as psoriasis, diabetic retinopathy, thrombosis, and inflammatory disorders including arthritis and atherosclerosis and cancer (10, 11). It has been proposed that TLR4 contributes to these diseases through *inflammation-induced angiogenesis*. The recent association between bacterial infections and atherosclerosis has intensified the search for the biological functions of TLRs especially TLR4 in blood vessel formation (12). The exact mechanism needs to be elucidated.

Angiogenesis is the normal process required for the development of an extensive vasculature. With its over 60 trillion endothelial cells, the vascular network is the first and the largest organ to develop in the human body (13). It mainly occurs during embryonic development. In adults, angiogenesis is a highly regulated process only occurring during the retinal development, in the adult intestinal villi and in the female reproductive organs (14). The postnatal angiogenesis may take place through one of the two possible mechanisms; (1) vasculogenesis – the *de novo* generation of blood vessels from endothelial progenitor cells (EPCs) or mesoderm and more commonly (2) angiogenesis, which is the sprouting/branching of the pre-existing blood vessels – together they are called neoangiogenesis. Angiogenesis is a highly complex series of sequential events orchestrating various molecular events involving multiple cell populations, cytokines, and chemokines. It takes place in two important steps; (1) formation of a nascent vascular network and (2) its subsequent maturation. The degradation of extracellular matrix (ECM) allows the sprouting of EPCs from old vessel into an avascular space and differentiation into nascent vasculature under the influence of pro-angiogenic factors. The maturation process involves the recruitment of supporting cells (mural cells) and vessel remodeling. Mural cells include vascular smooth-muscle cells (VSMC) in arteries, arterioles, and veins; pericytes in capillaries (15, 16). They provide structural integrity to the developing vasculature and may also interact with the endothelial cells, through paracrine signaling. Pro-angiogenic factors such as the vascular endothelial growth factor (VEGF); the basic fibroblast growth factor (bFGF); the transforming growth factor beta (TGF-β); the platelet-derived growth factor (PDGF); the tumor necrosis factor alpha (TNF-α); the insulin-like growth factor-1 (IGF-1); the monocyte chemotactic protein (MCP)-1; interleukin (IL)-6 and 8 all help in the recruitment of cells, ECM degradation, and with vessel development and maturity (14). An important empirical role played by TLR4 in the lymphocytic activation, recruitment, and release of cytokines is evident in TLR4-deficient mice. Such mice are reported to display significantly impaired expression of pro-inflammatory cytokines after reperfusion triggered by retinal ischemia injury (17). The process of lymphangiogenesis was shown to be affected in TLR4-deficient mice through lack of macrophage recruitment by TLR4^+^ lymphatic endothelial cells (LEC) (7).

As one of the two main sources of cytokines, macrophages play a critical role in the leukocyte trafficking and the postnatal angiogenesis. TLR4-mediated LPS-activated macrophages have been shown to be an important source of pro-angiogenic factors. Accumulating evidence shows that antigenic stimulation and the surrounding cytokine environment can have profound effects on the activation status and the functional capabilities of macrophages. Although there are various schools of thought regarding the macrophage activation status, here, we focus on two; the M1 and M2 phenotypes. The classical activation or M1 phenotype of macrophages contributes substantially toward anti-microbial immune responses via the production of pro-inflammatory cytokines such as IL-6, IL-8, IL-12, inducible nitric oxide synthase (iNOS), and interferons (IFNs) (18) (Figure 1). The alternate activation of macrophages may lead to the M2 phenotype, which is reported to be involved in the wound repair and fibrosis by contributing toward angiogenesis through the VEGF production (19). The strong mitogenic effect on the endothelial cells and the induction of vascular permeability are the pro-angiogenic effects, which makes VEGF the most potent simulator of angiogenesis. In murine macrophages and other TLR4^+^ cell populations, a strong synergism is reported to significantly influence the production of VEGF. Endotoxins (including LPS) together with the growth factors and cytokines such as IFN-γ, TGF-β, IL-1, and IL-6 have been implicated in a significant augmentation in VEGF levels (20–24). In this regard, the synergism reported between TLR4 and adenosine receptor 2A (A_2A_R) in the murine macrophages (M2) is noteworthy (Figure 1) (25). Adenosine receptor signaling plays an important role in inflammation. Adenosine is produced by many different cell types and is elevated in conditions such as hypoxia, ischemic conditions, and stress. So far, four adenosine receptors have been reported, i.e., the A_1_, A_2A_, A_3B_, and A_3_ receptors (26). The synergistic effect of A_2A_R is not restricted to TLR4, but TLR2, 7, and 9 also lead to high VEGF production in the presence of adenosine signaling (22). Both TLR4 and A_2A_R were shown to signal through hypoxia inducible factor (HIF)-1α and hypoxia response element (HRE) (27). Although the TLR4 along with its co-receptors are known to be expressed on the endothelial cells, it is not yet known whether the endothelial cells share the synergistic effect of TLR4 with A_2A_R. The transcriptional expression of A_2A_R has been reported on the endothelial cells; however, there are limited number of studies in this context. Many groups have demonstrated potent endothelial responses to LPS *in vitro* (28–32). However, there are reports supporting the *in vivo* role of LPS in postnatal angiogenesis. A study conducted in murine tumor model (metastatic) demonstrated the pro-angiogenic effects of LPS. The LPS-induced growth and metastasis of 4T1 experimental lung metastases model was shown to take place through increased angiogenesis, vascular permeability, and tumor cell migration (33). The LPS-mediated angiogenic effects can be reversed through TLR4 downregulation. While studying the anti-inflammatory affects of a compound known as *Baicalein*, its anti-angiogenic effects were shown to be carried out through the downregulation of TLR4 and its downstream mitogen-activated phosphate kinase (MAPK) pathway (34).

**Figure 1:**
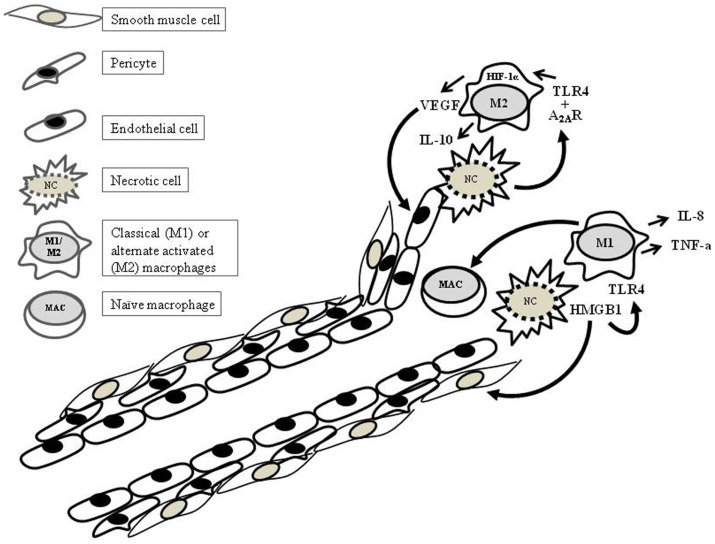
**TLR4 in postnatal angiogenesis**.

The ubiquitous and abundantly expressed DAMPs are often found in association with different anomalies. One such commonly expressed protein is high mobility group chromatin protein B1 (HMGB-1). It is a nuclear DNA binding protein released by injured or necrotic cells. Resting, non-activated inflammatory cells, such as monocytes or macrophages, contain HMGB-1 in their nuclei. When these cells are activated by LPS or inflammatory cytokines, HMGB-1 translocates in the cytoplasm, undergoes acetylation, and is exocytosed. It is evident that excreted HMGB-1 acts like a pro-inflammatory cytokine, therefore, HMGB-1 can be regarded as a signal of tissue injury and a mediator of inflammation (35). Macrophage-derived HMGB-1 has been shown to increase the endothelial cell proliferation, sprouting, and chemotaxis by stimulating the migration of adherent cells, such as fibroblasts and smooth-muscle cells. In a recent study, HMGB-1-TLR4 signaling was reported to be an important mediator in retinal neoangiogenesis in an oxygen-induced retinopathy murine model (36). HMGB-1 is an important marker for tumor endothelial cells and was shown to be necessary for the sustained expression of pro-angiogenic genes. A positive feedback mechanism has been suggested for the HMGB-1 expression and that of its cognate receptors, i.e., TLR4 and receptor for advanced glycation end products (RAGE) on the endothelial cells. Thus HMGB-1 may prove to be a promising target for interfering with cancer-related angiogenesis (37). However, there is some disagreement in relation to the HMGB-1 as an endogenous ligand for TLR4. The lack of an LPS-free *in vitro* system makes it difficult to study the signaling resulting exclusively from the TLR4-ligands other than LPS. Even small traces of LPS can upregulate TLR4 and can affect the interpretation of results.

Ischemic diseases are one of the major causes of morbidity and mortality. Treatment of such disorders requires angiogenesis. It is therefore the prime goal of *therapeutic angiogenesis* to achieve this. However, the close association between angiogenesis and inflammation presents an obstacle to the success of the therapy. Most of the pro-angiogenic factors are also pro-inflammatory. Therefore, the reperfusion of ischemic tissues often results in injury due to the microvascular dysregularities and inflammation (edema) associated with it. The activated endothelial cells lead to an imbalance between oxygen radicals and nitric oxide causing the release of inflammatory mediators (38, 39). The TLR4-deficient mice have been a valuable tool for studying the role of TLR4 in tissue-related ischemia–reperfusions *in vivo*. A recent study reported the role of TLR4-mediated responses contributing to the oxygen-induced neovascularization in ischemic neural tissue (retina). The TLR4-dependent responses, proposed to be mediated through HMGB-1 release in the ischemic neural tissue were found to be impaired in TLR4-deficient mice, revealing an important angiogenic role of TLR4 in neural tissues (36). On the other hand, there are several studies highlighting the inflammatory role of TLR4 in various reperfusion–ischemic models in tissues such as liver, lung, and intestine. Most of these studies showed reduced inflammation in relation to the injury induced by the reperfusion of various organs after a period of ischemia in TLR4-deficient mice, thus, highlighting the inflammatory role of TLR4 in reperfusion-related injury models, without significant compromise in angiogenesis (40–43). Considering these reports, the dual role of TLR4 in angiogenesis and inflammation comes to light, which seems to be governed by an intricate balance between the inhibitory or stimulatory factors that may be tissue-specific. Nevertheless, TLR4 remains a promising target for suppressing the undesired and prolonged inflammatory responses. In this regard, various synthetic and plant-derived therapies are currently being tested. TLR4-blocking through small molecule inhibitors and antibodies are being evaluated in pre-clinical trials for their efficacy in various inflammatory conditions. Novimmune is a humanized counterpart of rat anti-TLR4 monoclonal antibody; 1A6, found to reduce inflammation in a murine colitis model. It is undergoing pre-clinical evaluation for the treatment of the inflammatory bowel diseases (44–46). Various plant-derived drugs such as wogonoside and celastrol have shown promising results against TLR4-mediated LPS-induced angiogenesis in pre-clinical drug testing (47, 48).

In conclusion, it can be said that the close association between inflammation and angiogenesis makes the therapeutic modulation of TLR4 somewhat challenging and can lead to potential side effects. Therefore, the fine tuning of TLR4 and its associating proteins is required in order to circumvent the undesired inflammatory or angiogenic responses associated with TLR4 targeting in various pathologies. For that purpose, further insight into its *in vivo* networking and the effects of TLR4 targeting in various pathologies through the use of closely related animal disease models is required.

## Conflict of Interest Statement

The author declares that the research was conducted in the absence of any commercial or financial relationships that could be construed as a potential conflict of interest.
